# Correspondence

**Published:** 1871-04

**Authors:** 


### Correspondence.

*Editor of the Buffalo Medical and Surgical Journal:*

DEAR SIR,—The following paragraph is quoted from an article signed John J. Burke, M. D., coroner, published in several of our daily newspapers:

"I asked to see Dr. Hauenstein's certificate. That document stated that Koch died from inflammation of the lungs. True, the right lung was nearly gone. But why did this physician, after telling the family that it was evident the patient would die—a fact evident at the day of his birth—allow Koch to walk all around the neighborhood, and frequent the saloons, and come home intoxicated, while under his care? Why was his son ignorant of the near approach of death and of deceased having made a will inside of five days? The deceased came home from some saloon, got a pail of water and went out in the yard to give it to some pigs—came back into the house and fell under the kitchen table, dead. The physician who made the *post-mortem* pronounced his death to have been brought about by heart disease, accelerated by intemperance, in which the jury concurred. I say that the verdict was correct, and that deceased came to his death by the 'stereotyped disease of the heart.'"

Now, the coroner never saw any certificate stating the cause of death of John Koch, for it was in my possession until a few days ago. The coroner could not know that "the right lung was nearly gone," because no *post-mortem* dissection was made at the time of the inquest that brought to view the lungs. My duty to the patient was neither that of keeper nor of nurse. He never asked me to allow him to get intoxicated; and I have the best of evidence that he did not get intoxicated at all while under my care. The son, personally, with horse and buggy, went for a notary pnblic, and was present when the will was executed. The deceased never got a pail of water during his illness to give it to some pigs: the pigs were creatures of his imagination—the family never kept any pigs; and the deceased did not leave the premises for three days previous to his death. Lastly, what evidence had the coroner that deceased came to his death from "disease of the heart," when he never saw, heard, or felt, the deceased's heart? For, I repeat, no *post-mortem* dissection was made at or before the time of the inquest to bring to view the heart. By which of the five senses was he enabled to determine the morbid condition of the heart? Although charitably inclined, I am not even permitted to allow him the advantage of his olfactory acuteness. It was not by means of any sense, but by means of nonsense, that the conclusion was arrived at. The son of deceased being interrogated by the coroner of what disease the Doctor said his father had died, to which the son answered, "I don't know, he never told me." Well, says the coroner, "I guess we call it heart disease." Well done, *honest* and *truthful* servant!

Respectfully Yours,

JOHN HAUENSTEIN.

NOTE.--The newspaper article referred to, and quoted from, by Dr. Hauenstein, has appeared to us to be too low, vindictive, untruthful, undignified and unprofessional to permit reply. Accusing us, and a student, of having caused death by chloroform, and of having had a suit for malpractice, appears as defence for a great many unnecessary and wholly unjustifiable *post-mortem* examinations in cases of death from well known, and often times, long standing disease—where there was not even a suspicion of crime. If our young coroner (coroner plainly enough) had added to the intimation of our causing death by chloroform, that the *post-mortem* and microscopic examination showed complete fatty degeneration of the heart, and that, in our malpractice suit, it was the unanimous testimony of the medical profession, called as witnesses, *on both sides*, "that the practice was correct, and the result as good as could be expected," resulting in our entire and complete acquital; and then added that these cases had been reported in our own Journal as items of general professional interest, as well as of personal and professional pride, he would have manifested a better spirit in his defence. It would hardly seem possible that a Physician could omit, in such connection, that part in which all the members of the *profession* have a common interest and a common pride But his article is in every respect unworthy reply, and we leave to his associates to judge if they are safe in his company.—ED.

